# Myxoma Immediately above the Junction of the Inferior Vena Cava and the Right Atrium: A Rare Cause of Budd-Chiari Syndrome 

**Published:** 2016-07-06

**Authors:** Vahid Mohammad Karimi, Amir Anushiravani, Mohammad Hossein Dabbaghmanesh, Massood Hosseinzadeh, Ali Reza Rasekhi, Mahmoud Zamirian, Amir Anushiravani

**Affiliations:** Nemazee Hospital, Shiraz University of Medical Sciences, Shiraz, Iran.

**Keywords:** *Myxoma*, *Budd–Chiari syndrome*, *Vena cava, inferior*

## Abstract

The Budd–Chiari syndrome (BCS) is a rare disorder caused by the obstruction of the hepatic veins or the inferior vena cava (IVC) at the suprahepatic level. This syndrome is developed by either hepatic vein thrombosis or mechanical venous obstruction and leads to centrilobular hepatic congestion with the subsequent development of fibrosis and cirrhosis. Intracardiac tumors have been rarely reported as a cause of the BCS. These tumors usually originate from the atrial septum. Very rarely, they arise either from the junction of the IVC and the right atrium or from the Eustachian valve. There are a few case reports in the literature where atrial tumors have caused the BCS. In these cases, the tumors were malignant, and the patients died shortly after being diagnosed.

We describe a 71-year-old female patient who presented with a 3-month history of abdominal pain and protrusion. On physical examination, blood pressure and pulse rate were normal. Jugular venous pressure was about 10 cm. Cardiac examination revealed a systolic murmur, grade IV/VI, in the left sternal border without radiation. Echocardiography showed a large mass (about 6×4 cm) in the right atrium with close contact to the origin of the IVC, obstructing it. Cardiac magnetic resonance imaging, with and without gadolinium, also confirmed the diagnosis. The patient underwent surgery, and the myxoma was removed. The tumor was a large solid mass, 5×4 cm in size, which originated immediately above the entrance of the IVC. The patient is in good condition 1 year afterward. We emphasize that atrial myxomas should be considered in the differential diagnosis of tumors that cause chronic BCS.

## Introduction

The Budd–Chiari syndrome (BCS) is a rare disorder caused by the obstruction of the hepatic veins or the inferior vena cava (IVC) at the suprahepatic level. This syndrome is developed by either hepatic vein thrombosis or mechanical venous obstruction and leads to centrilobular hepatic congestion with the subsequent development of fibrosis and cirrhosis. Intracardiac tumors have been rarely reported as a cause of the BCS.^1^, ^2^ These tumors usually originate from the atrial septum.^3^ Very rarely, they arise either from the junction of the IVC and the right atrium or from the Eustachian valve. 

We found a few case reports in the literature where atrial tumors have caused the BCS. In these cases, the tumors were malignant, and the patients died shortly after being diagnosed. We describe a patient with newly diagnosed BCS, which constitutes a rare presentation of a large right atrial myxoma originating immediately above the junction of the IVC and the right atrium.

## Case Presentation

**Figure 1:**
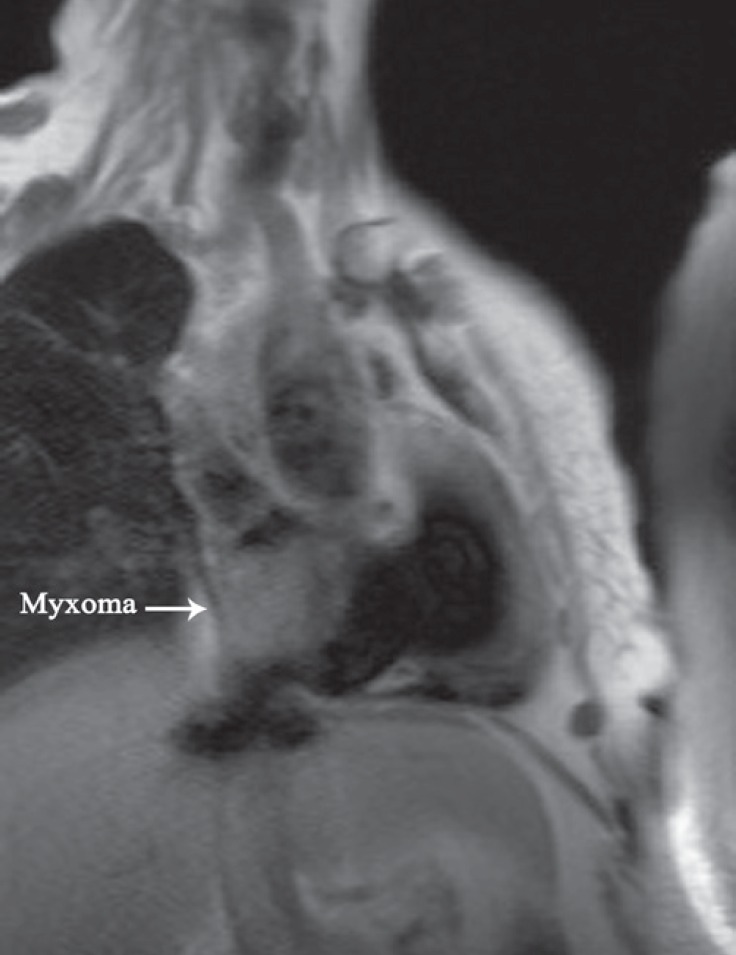
T2 cardiac magnetic resonance imaging, coronal view, shows that the right atrial myxoma (arrow) obstructs the entrance of the inferior vena cava.

A 71-year-old female patient presented to our hospital with a 3-month history of abdominal pain and protrusion. A stool examination was requested, which revealed occult blood. Colonoscopy revealed a diverticulum, which was subsequently resected from the transverse colon. Her complaint continued, however, and she developed lower extremity edema and an increase in abdominal protrusion. On physical examination, blood pressure was 130/90 mm Hg and pulse rate 90 bpm. Jugular venous pressure was about 10 cm. Cardiac examination revealed a systolic murmur, grade IV/VI, in the left sternal border without radiation. The abdomen was soft without organomegaly, but the right upper and lower quadrants had mild tenderness. Shifting dullness was positive, and lower extremities also had 3+ pitting edema. ECG illustrated normal sinus rhythm and axis without any ischemic change. Chest X-ray showed an increased cardiothoracic ratio. Laboratory data revealed white blood cells of 6800/mL, hemoglobin of 13.2 mg/dL, platelet count of 140000/mL, aspartate transaminase of 29 U/L, alanine transaminase of 50 U/L, alkaline phosphatase of 240 U/L, total bilirubin of 1.52 mg/dL, direct bilirubin of 0.5 mg/dL, albumin of 3.2 mg/dL, prothrombin time of 16 seconds, partial thromboplastin time of 37 seconds, and international normalized ratio (INR) of 1.7. Abdominal tap indicated high-gradient ascites. The patient had an albumin level of 0.4 mg/dL. Our first step was to approach this high-gradient ascites, so we performed an abdominal sonography, which showed the normal size and echogenicity of the liver and spleen. Color Doppler sonography revealed normal portal, hepatic, and splenic veins without thrombosis, but the IVC was dilated. Echocardiography showed a large mass (about 6 × 4 cm) in the right atrium with close contact to the origin of the IVC, obstructing it. Cardiac magnetic resonance imaging (MRI) also confirmed the diagnosis (Figure 1).

T2 coronal MRI revealed a 4.8 × 3.9 cm hypersignal mass in the right atrium, immediately above the junction of the IVC and the right atrium. The IVC and hepatic veins were dilated.

**Figure 2:**
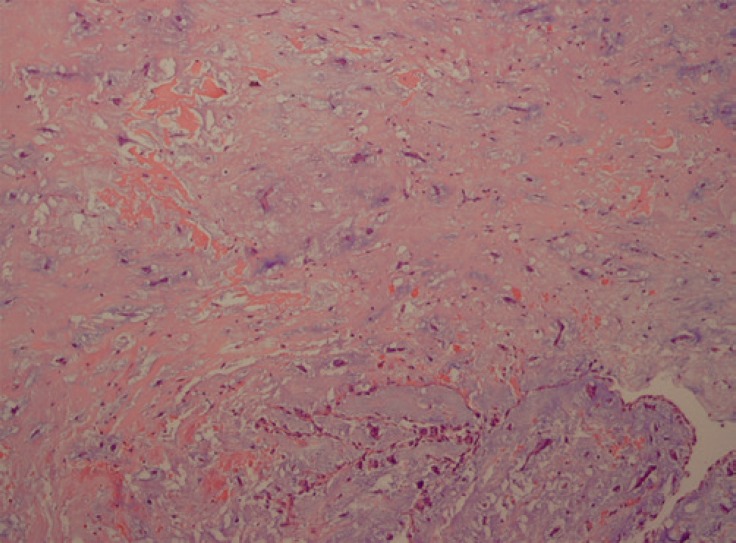
Microscopic view from the myxoma tissue in low-power field shows a low cellular neoplastic tissue with a myxoid background (H&E × 100).

**Figure 3:**
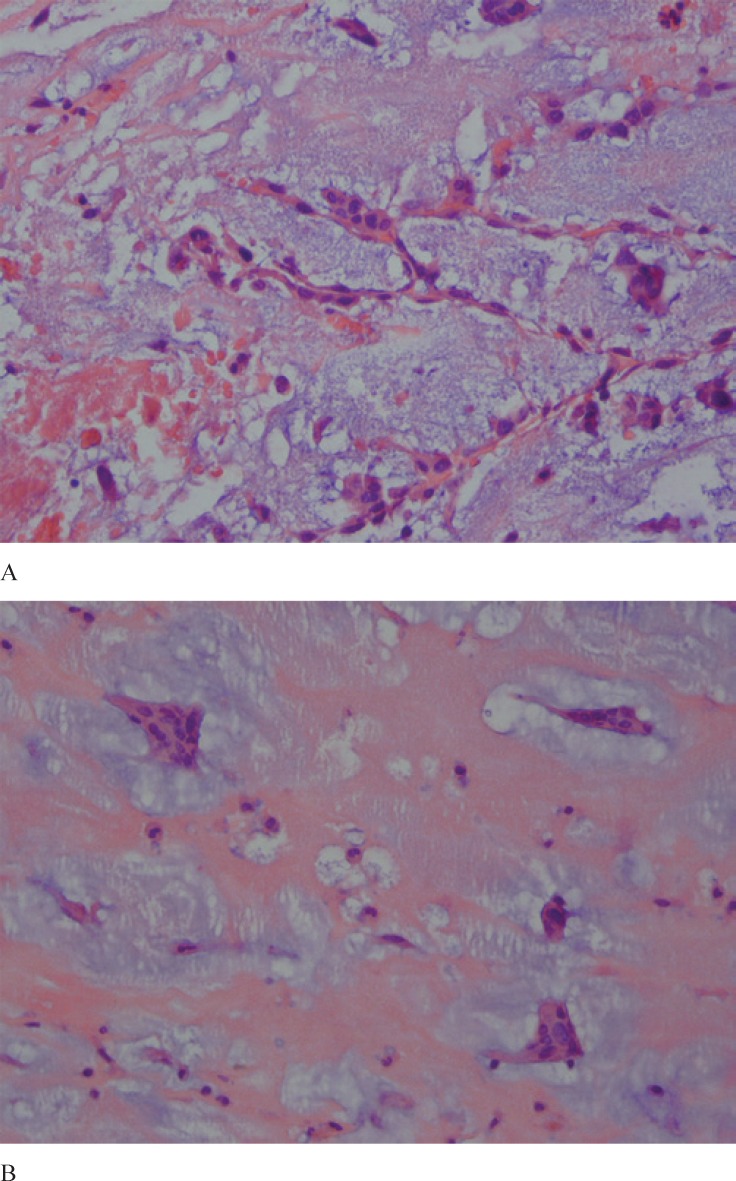
High-power field microscopic views (A & B) of the myxoma tissue show stellate cells in a myxoid background (H&E × 400).

The patient underwent surgery, and the myxoma was removed. The tumor was a large solid mass, 5 × 4 cm in size, which originated just above the entrance of the IVC. Pathology reported a low cellular neoplastic tissue with a myxoid background in low-power field (H&E × 100) (Figure 2). High-power field showed stellate cells in a myxoid background (H&E × 400) (Figure 3).

## Discussion

The BCS is a rare disorder characterized by a hepatic venous outflow obstruction anywhere between the small hepatic veins and the entrance of the IVC into the right atrium. The BCS is classified as primary, when the obstruction of the hepatic venous outflow tract results from an endoluminal venous lesion (thrombosis or congenital webs) or secondary, when the cause of the obstruction is an extrinsic compression or nearby invasion by an abscess, cyst, or benign or malignant tumor.^4^^, ^^5^ Common risk factors are hypercoagulable states associated with polycythemia vera,^6^ myeloproliferative disorders, paroxysmal nocturnal hemoglobinuria, antiphospholipid syndrome, and defects in the coagulation cascade such as protein C and S deficiency or factor V Leiden mutations. The BCS can have an acute or chronic presentation. The acute form of the BCS may present with fulminant hepatic failure, encephalopathy, ascites, jaundice, tender hepatomegaly, and hepatorenal syndrome.^5^ Chronic BCS is characterized by a history of vague complaints for about 6 months and the onset of ascites, presenting as portal hypertension. Laboratory tests show features of chronic liver disease such as low albumin, prolonged prothrombin time, and near-normal aminotransferase. Our patient’s presentation was in favor of the chronic form of the BCS. The diagnosis of the BCS is often made by noninvasive imaging modalities. Ultrasonographic evaluation of the liver and its vasculature is useful for this purpose inasmuch as it can reveal the location of the obstruction. Nonvisualization of the hepatic veins, areas of stenosis, collateral veins, and proximal dilatation can suggest the BCS. An altered or absent hepatic venous flow may increase diagnostic accuracy.^7^ Computed tomography is also useful in evaluating the patency of the hepatic veins and the liver. Echocardiography is a useful diagnostic imaging modality in that it may show valvular stenosis or prolapse of the tumor through the atrioventricular valve. MRI and computed tomography are also useful. The differential diagnosis of a mass in the right atrium includes myxoma, thrombus, a primary malignant cardiac tumor, and metastasis.^8^ Cardiac thrombi are the most frequent cardiac masses, and they are mainly caused by impaired wall motion or arrhythmias. However, thrombi in the right atrium are very rare. MRI is a sensitive imaging modality for detecting thrombi. In our patient, MRI showed a large mass just above the junction of the IVC and the right atrium. Cardiac tumors are rarely associated with the BCS. Tumors may cause the BCS either by direct invasion or by external pressure.^1^^, ^^2^ Primary tumors of the heart account for only 0.3% of cardiac tumors.^9^ We found a few case reports in the literature where atrial tumors have caused the BCS. In these cases, the tumors were malignant, and the patients died shortly after diagnosis.^1^ Nonetheless, in our patient, histopathologic examination showed features typical of myxomas. We found only 11 other cases in the literature reporting a myxoma originating from the IVC.^2^^, ^^10^^-^^19^ In Table 1, we compare our patient’s presentation with previous reported cases.

**Table 1:** Presentations and sites of attachment in the reported cases of cardiac myxomas

Study	Presentation	Site of Attachment
Devig PM et al.^10^ (1980)	Dyspnea	Junction of IVC and right atrium
Cujec B et al.^2^ (1987)	BCS	Eustachian valve
Bortolloti U et al.^11^ (1990)	Dyspnea and Edema	Suprahepatic IVC
Penta de Peppo et al.^12^ (1992)	Dyspnea	Junction of IVC and right atrium
Teoh KH et al.^13^ (1993)	Paroxysmal atrial tachycardia	Eustachian valve
Bonde P et al.^14^ (2002)	Colonic polyposis	Eustachian valve
Elmusa K et al.^15^ (2004)	Dyspnea and chest pain	Suprahepatic IVC
Anagnostopoulos GK et al.^16^ (2004)	BCS	Junction of IVC and right atrium
Juneja MS et al.^17^ (2006)	Dyspnea and syncope	Suprahepatic IVC
Ozer N et al.^18^ (2009)	Asymptomatic	Upper border of IVC
Darwazah AK et al.^19^ (2011)	Asymptomatic	Junction of IVC and right atrium

IVC, Inferior vena cava; BCS, Budd–Chiari syndrome

Clinical manifestations vary from exertional dyspnea to the BCS. A review of these myxomas yielded only 2 cases as the BCS.

Atrial myxomas are the most common primary tumors of the heart. They account for 35% to 50% of primary cardiac tumors and are usually located in the left atrium near the fossa ovalis. Only 20% of myxomas arise from the right atrium.^20^

## Conclusion

We herein introduced a rare presentation of the BCS caused by a right atrial myxoma originating immediately above the IVC entrance. It is advisable that atrial myxomas be considered in the differential diagnosis of tumors that cause chronic BCS.

## Notes:

***This paper should be cited as: ***Mohammad Karimi V, Anushiravani A, Dabbaghmanesh MH, Hosseinzadeh M, Rasekhi AR, Zamirian M, Anushiravani A. Myxoma Just above the Junction of Inferior Vena Cava and Right Atrium: A Rare Cause of Budd-Chiari Syndrome. J Teh Univ Heart Ctr 2016;11(3):139-142.
